# Near-infrared light harvesting of upconverting NaYF_4_:Yb^3+^/Er^3+^-based amorphous silicon solar cells investigated by an optical filter

**DOI:** 10.3762/bjnano.9.260

**Published:** 2018-10-31

**Authors:** Daiming Liu, Qingkang Wang, Qing Wang

**Affiliations:** 1 College of Physics and Lab of New Fibre Materials and Modern Textile Growing Base for State Key Laboratory, Qingdao University, Qingdao 266071, P.R. China; 2 Key Laboratory for Thin Film and Microfabrication Technology of Ministry of Education, School of Electronic Information and Electrical Engineering, Shanghai Jiao Tong University, Shanghai, 200240, P.R. China; 3 Institute of NanoEngineering, School of Material Science and Engineering, Shandong university of Science and Technology, Qingdao, 266590, P.R. China

**Keywords:** filter, light harvesting, solar cells, upconverting

## Abstract

The wastage of near-infrared light seriously restricts the photoelectric conversion efficiency of hydrogenated amorphous silicon (a-Si:H) thin film solar cells. Spectral upconversion is of great significance in reducing the wastage. Herein, the upconverting compound NaYF_4_:Yb^3+^/Er^3+^ was synthesized via a hydrothermal method. SEM and XRD results revealed the morphology and a phase transition from cubic to hexagonal NaYF_4_. Photoluminescence spectra indicated that the hexagonal NaYF_4_:Yb^3+^/Er^3+^ nanorods convert near-infrared light of 980 nm to the visible light with wavelength peaks at 654, 541 and 522 nm. Hence, the upconverting rods were incorporated in a polymethylmethacrylate (PMMA) layer on the rear side of a-Si:H solar cell. Under AM1.5 solar irradiation, a facile optical filter was used to scrutinize the effect of upconversion on the cell performance. Compared with a bare cell, the NaYF_4_:Yb^3+^/Er^3+^-based a-Si:H cell exhibited an 25% improved short-circuit current and an appreciable improvement of the near-infrared response of the external quantum efficiency. Moreover, because the size of the nanorods is comparable to the wavelength of visible light, the rods effectively scattered light, thus enhancing the visible light harvesting.

## Introduction

One potential way to meet the increasing energy consumption requirements lies in the utilization of solar energy. Solar cells are expected to play an important role in relieving the global energy crisis. Among solar cells, the hydrogenated amorphous silicon (a-Si:H) thin-film solar cell is one of the most promising candidates due to its high inherent absorption coefficient, short charge-carrier diffusion length and low production cost [1]. Films of a-Si:H with a wide bandgap of ca. 1.75 eV have a high absorption in the visible range; however, they are almost transparent for near-infrared radiation (NIR, 700–2500 nm), which constitutes 52% of the energy of the entire solar spectrum [2–3]. The transmittance of NIR light is one of the major energy-loss mechanisms for conventional a-Si:H solar cells [4–5]. This energy loss can be substantially reduced through spectral upconversion (UC) [6]. Spectral UC describes nonlinear anti-Stokes optical processes that can convert two (or more) NIR photons to a visible photon. In terms of its application in solar cells, UC is expected to convert the sub-bandgap NIR light to above-bandgap visible light. The photons generated during upconversion are then absorbed by photoactive semiconductors to generate electron–hole pairs. This means that UC can broaden the absorption spectrum and enhance the photoelectric conversion efficiency of solar cells. Based on a detailed balance model, the upper limit of the photoelectric conversion efficiency of silicon solar cells, equipped with an ideal upconverter, is calculated to be 47.6% [7], which is far above the Shockley–Queisser limit of ca. 32%. Thus, UC has great potential for the efficiency enhancement of solar cells.

Up to now, sodium yttrium fluoride (NaYF_4_) doped with trivalent lanthanide ions, such as Yb^3+^, Gd^3+^ and Er^3+^, is the most promising material for upconversions [8]. It has emission bands in the visible spectrum, where solar cells have the high internal collection efficiency [9]. Moreover, the absorption band of this material lies in NIR range, not deteriorating the visible light response of solar cells. Other important thing is that such upconverting materials with nanoscale dimensions can be easily re-dispersed into a binding/adhesive agent [10]. Because the upconverter is supposed to use the transmitted light, it can be easily implemented on the rear side of solar cells, separated from the actual physics of the operating device [6].

Although the idea of using UC for improving the cell performance is obvious, relatively little work has been done so far [11]. Due to the relatively weak intensity of NIR light and the low conversion efficiency, the effect of UC on the cell performance is weak and difficult to identify [12]. In order to better measure the UC effect, some experiments have been performed on solar cells under laser illumination. For example, a-Si:H cell with NaYF_4_:Yb/Er/Gd upconverters showed a 16-fold to 72-fold improvement of the photocurrent under 980 nm laser light [13]. An enhancement of 10 mA/cm^2^ in a NaYF_4_:Yb/Er-based a-Si:H cell was measured under illumination with a 980 nm diode laser (10 mW) [14]. The usage of laser undoubtedly enhances the illumination intensity and thus magnifies the UC effect; however, it does not imitate the actual solar irradiation in practical devices.

In the present work, the upconverting NaYF_4_:Yb^3+^/Er^3+^ nanorods were synthesized thorugh a hydrothermal method and their UC effect on NIR light harvesting in a-Si:H solar cell was scrutinized by using a facile optical filter. Photoelectric performances of a-Si:H solar cells with and without UC were compared under AM1.5 solar irradiation.

## Results and Discussion

**Figure 1:**
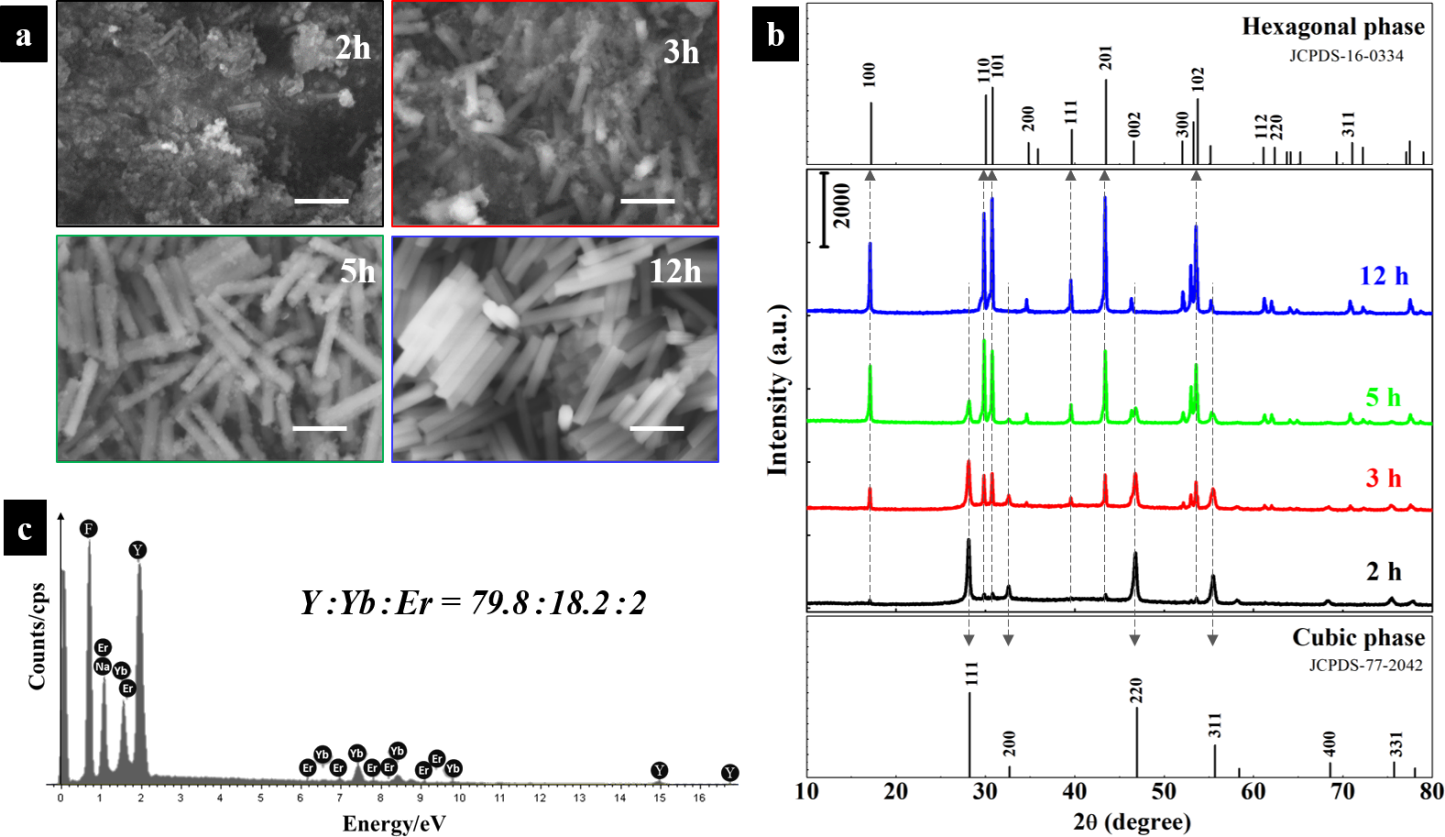
(a) FE-SEM morphologies of NaYF_4_:Yb^3+^/Er^3+^ as a function of the reaction time (2, 3, 5 and 12 h). All scale bars are equal to 1 μm. (b) XRD patterns of powder samples, comparing to the standard cards of hexagonal β-NaYF_4_ (JCPDS-16-0334) and cubic β-NaYF_4_ (JCPDS-77-2042). (c) EDX patterns of NaYF_4_:Yb^3+^/Er^3+^ as a function of the reaction time of 12 h.

Field-emission scanning electron microscopy (FE-SEM) was used to investigate the sample morphologies over the course of the reaction. In Figure 1a, the as-produced powder over the first 2 h is a mixture of predominantly nanoparticle aggregates with some hexagonal nanorods. As the reaction continues, the fraction and the dimensions of the nanorods are apparently increased. After 12 h, the sample is made up of hexagonal nanorods with well-defined and smooth facets. No nanoparticles are observed and the UC nanorods exhibit a monodisperse size distribution. On average, they are ca. 200 nm in diameter and ca. 1.5 μm in length. According to the X-ray diffraction (XRD) patterns in Figure 1b, with increasing reaction time, reflections of the hexagonal β-NaYF_4_ phase (JCPDS-16-0334) increase in intensity, while the reflections belonging to the cubic β-NaYF_4_ phase (JCPDS-77-2042) weaken. We can infer that the nanoparticles have a cubic crystal structure, while the nanorods are hexagonal. XRD patterns agree well with FE-SEM results. The cubic-to-hexagonal phase transition is completed after 12 h. In Figure 1c, energy-dispersive X-ray (EDX) analysis confirms the doping with Yb and Er. The molar ratio of Y/Yb/Er in the hexagonal phase NaYF_4_:Yb^3+^/Er^3+^ was determined to be 79.8:18.2:2. It is extremely close to the stoichiometric ratio of 80:18:2 of the most efficient UC materials [15–16].

**Figure 2:**
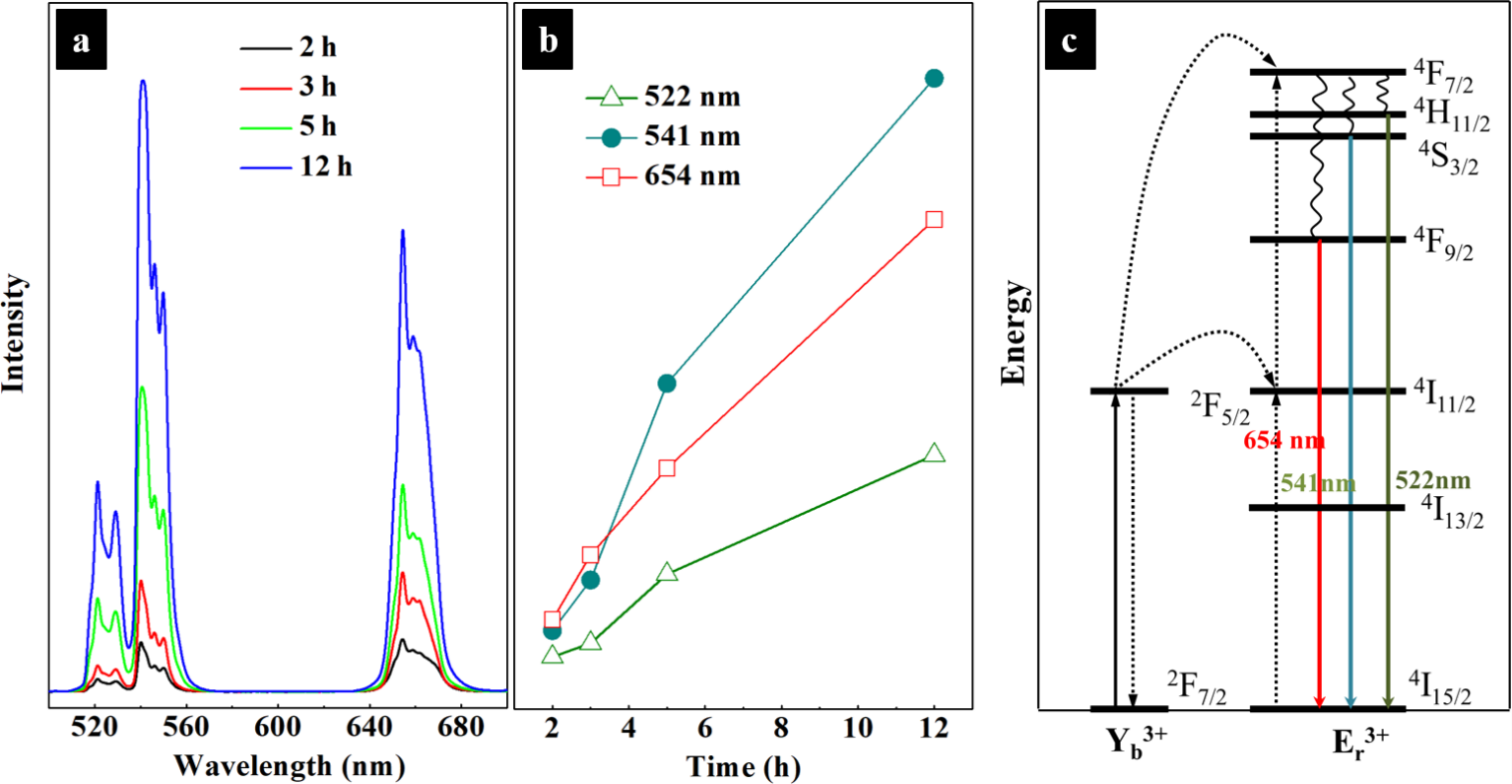
(a) Photoluminescence spectra of NaYF_4_:Yb^3+^/Er^3+^ samples under 980 nm laser illumination with a power of 60 mW; (b) peak intensities at 522, 541, and 654 nm as a function of the reaction time. (c) energy transfer mechanisms for the Yb^3+^–Er^3+^ couple under 980 nm laser illumination.

Photoluminescence spectra of NaYF_4_:Yb^3+^/Er^3+^ powders from 500 to 700 nm are presented in Figure 2a. Three strong emission peaks at 522, 541, and 654 nm are observed. The intensity of each emission peak increases with increasing reaction time (Figure 2b), which is interpreted as an increase in the fraction of the hexagonal phase. The quantum efficiency of the hexagonal nanorods is about an order of magnitude higher than that of the cubic phase counterparts [17]. The photoluminescence of NaYF_4_:Yb^3+^/Er^3+^ is explained by energy transfer between Yb^3+^ and Er^3+^. As illustrated in Figure 2c, under 980 nm excitation with a power of 60 mW, Yb^3+^ ions firstly capture the energy of the photons and then transfer it to neighboring Er^3+^ ions in a ^4^I_11/2_ → ^4^F_7/2_ transition. After non-radiative relaxation to lower energy levels, the excited electrons return to the ground level (^4^I_15/2_) under emission of green or red photons [11,16].

**Figure 3:**
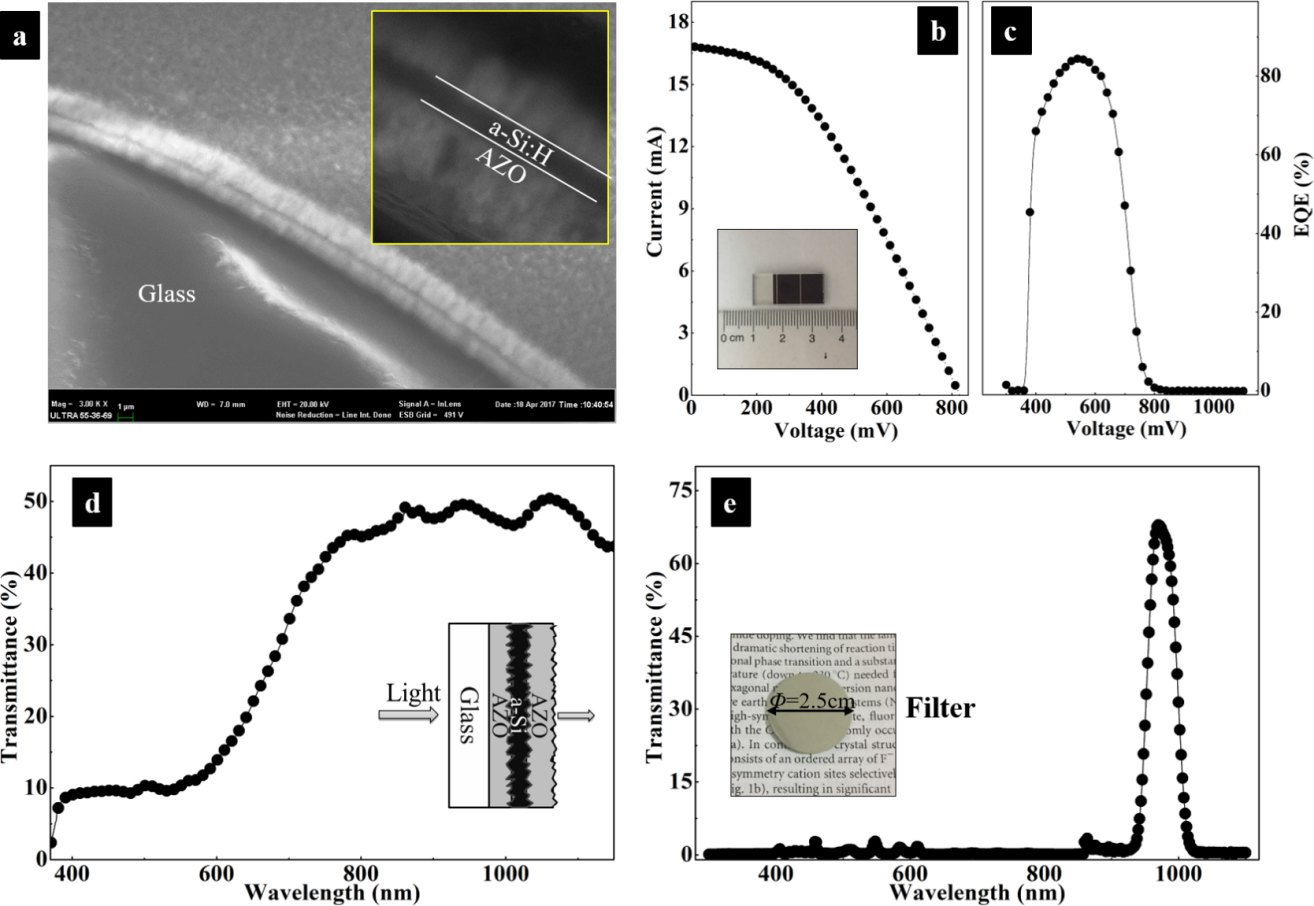
(a) FE-SEM images showing the multiple layers of a-Si:H solar cell; (b) current–voltage and (c) EQE curves of a-Si:H solar cell under AM1.5 solar irradiation. (d) transmittance spectrum of a-Si:H solar cell without reflector in 370–1100 nm; (e) transmittance spectrum of the optical filter and the inset optical photograph.

Hexagonal NaYF_4_:Yb^3+^/Er^3+^ nanorods can efficiently convert NIR light to red and green light, which is of great interest in broadening the absorption spectrum of a-Si:H solar cells. As a research object, an a-Si:H solar cell with multiple layers was prepared (Figure 3a). One a-Si:H layer of ca. 350 nm is sandwiched between two aluminum-doped zinc oxide (AZO) contacts. The thickness of the AZO contacts is approximately 1.5 μm. From Figure 3b, the photoelectric conversion efficiency of the a-Si:H solar cell is calculated to be 6.68% at the maximum power point (0.45 V, 12.45 mA). The inset shows a photograph with the active area of ca. 0.84 cm^2^. The external quantum efficiency (EQE) curve in Figure 3c reveals that almost no photocurrent is generated when the wavelength of light is longer than 800 nm. The transmittance spectrum of a-Si:H solar cell without back reflector in Figure 3d shows that nearly 50% of the incident light with wavelengths longer than 800 nm passes through the cell. Given the large proportion of transmittance loss in NIR range, the a-Si:H solar cell would benefit much from the incorporation of UC.

**Figure 4:**
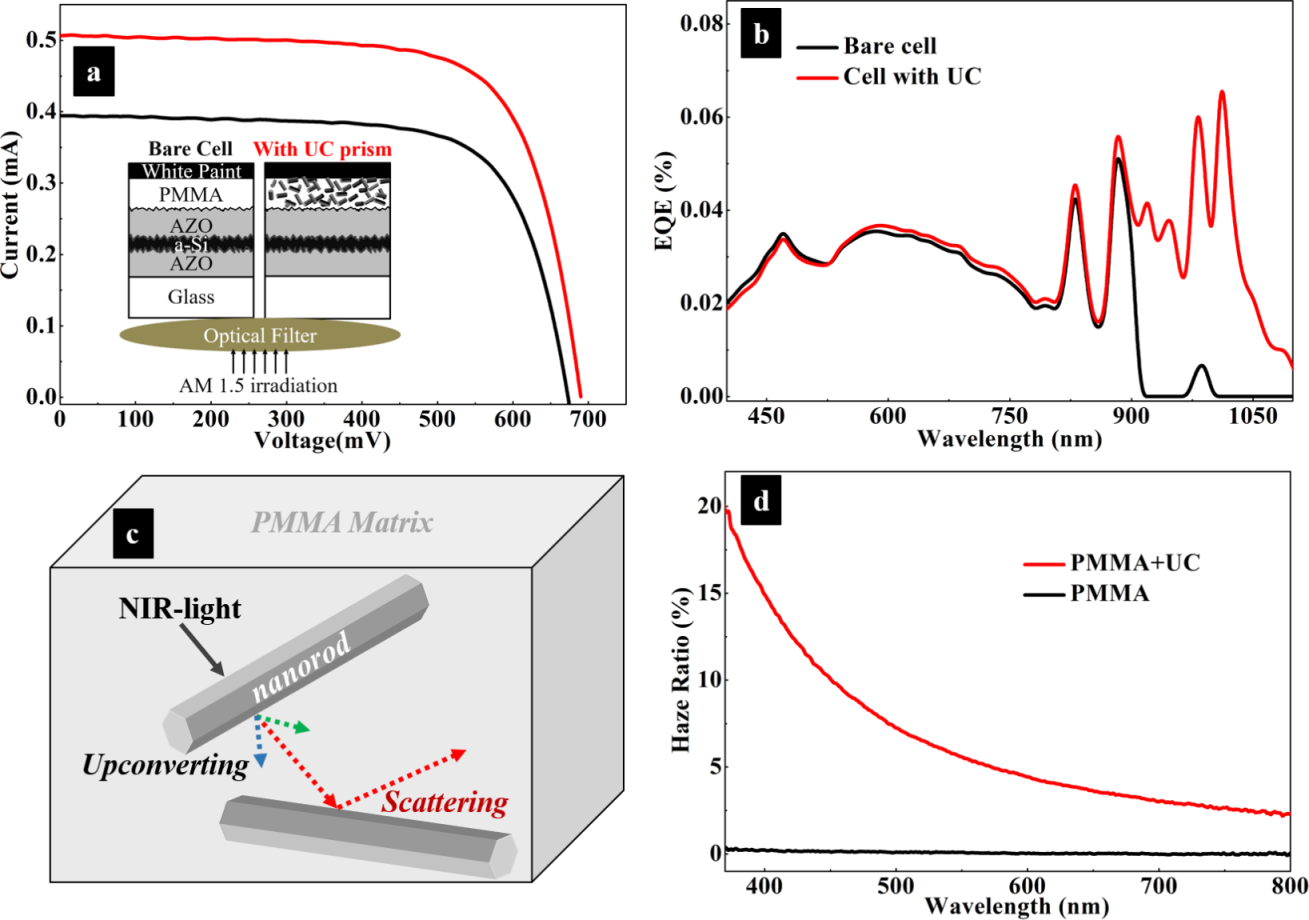
(a) Current–voltage curves and (b) EQE curves of an a-Si:H solar cell with an UC layer compared with the bare cell under AM1.5 solar irradiation through the optical filter. Inset in (a) is a schematic comparison of the two setups. Schematic diagram (c) of the UC and scattering effects of NaYF_4_:Yb^3+^/Er^3+^ prisms in a PMMA matrix. (d) Haze-ratio spectra of PMMA layers on glass with and without UC prisms. The thickness of the PMMA layer is ca. 4.5 μm.

Under solar irradiation, the relative weak UC effect is obscured by the strong primary absorption of the above-bandgap radiation. In order to distinguish the harvesting of upconverted photons from the primary absorption, a commercial optical filter is chosen to shield the above-bandgap light from the solar cell and only let certain sub-bandgap NIR light pass through. The transmittance spectrum of the optical filter is shown in Figure 3e. Its transmittance peak wavelength locates at 980 nm with a high transmittance of ca. 70%, and the full width at half maximum is ca. 50 nm. The optical filter was placed right above the a-Si:H solar cell, completely covering the photoactive area. Considering a practical use of the cell under sunlight, photoelectric measurements were conducted under AM1.5 solar irradiation. A bare a-Si:H solar cell without UC nanorods but with otherwise the same cell structure was used as reference. In Figure 4a, a short-circuit current of 0.4 mA is measured in the bare cell, while it is 0.5 mA in the cell with UC nanorods. The relative enhancement is 25%. The open-circuit voltage is also increased slightly. EQE curves in Figure 4b also confirm the improvement of spectral harvesting of the NIR light of 800–1100 nm. Around 0.05% in 900–1050 nm is obtained in a-Si:H solar cell with UC nanorods. In contrast, almost no response is found in the bare cell. This shows that the UC nanorods convert NIR light to the visible light that is reflected back into the cell and subsequently absorbed by the a-Si:H layer to increase the photocurrent and EQE.

Light scattering occurs on an object when its size is comparable to the wavelength of light [18]. Because the nanorods are comparable in size to the incident wavelength, they effectively scatter the upconverted and/or the incoming visible light, as illustrated in Figure 4c. Optical scattering can be measured by the haze ratio, which is defined as the ratio of diffusely transmitted light to total transmitted light [19–20]. The PMMA layer containing UC nanorods on glass exhibits a higher haze ratio in the range of 400–800 nm than a PMMA layer without nanorods (Figure 4d). The scattering effect changes the propagation direction of light and elongates the propagation path in the photoactive layer of the solar cell, thus enhancing the light harvesting of visible light.

## Conclusion

We have used hexagonal-phase NaYF_4_:Yb^3+^/Er^3+^ (18/2 mol %) nanorods on the rear side of a-Si:H solar cell and have demonstrated that the nanorods converted NIR light of ca. 980 nm to visible light of 522, 541, and 654 nm. Using an optical filter to block visible solar irradiation confirmed the improvement of the NIR light harvesting in a-Si:H solar cell. Compared with a bare cell, 25% of enhancement in short-circuit current and ca. 0.05% of EQE around 980 nm were obtained in the NaYF_4_:Yb^3+^/Er^3+^-based a-Si:H solar cell. The upconverting NaYF_4_:Yb^3+^/Er^3+^ nanorods, with feature sizes comparable to the wavelengths of visible light, also enhanced the harvesting through scattering. Our results explicitly demonstrated the feasibility of UC in NIR light harvesting for a-Si:H solar cells.

## Experimental

The NaYF_4_:Yb^3+^/Er^3+^ was prepared via a hydrothermal method based on a alcohol/water/oleic acid system [21]. Lanthanide chloride hexahydrate (RECl_3_·6H_2_O, RE = Y, Yb and Er; 99.99%), sodium hydroxide (NaOH, 98+%), ammonium fluoride (NH_4_F, 98+%) and oleic acid (OA, 90+%) were used as starting materials. 4.5 mL of NaOH solution (5 mol/L) was mixed with ethanol (15 mL) and OA (15 mL) under ultrasonic treatment. Next, to the resulting transparent solution was successively added 6 mL of RECl_3_ solution (0.2 mol/L, stoichiometric ratio) and 3 mL of NH_4_F solution (2 mol/L). Then, the mixture was transferred into a 75 mL Teflon-lined autoclave and heated at 200 °C for a given time. Finally, the as-prepared fine white powders were collected by centrifugation at 14000 rpm, washed with water and ethanol for several times, and dried at 70 °C. The a-Si:H solar cell with an intrinsic p–i–n conﬁguration was deposited on a 3 mm glass substrate by plasma-enhanced chemical vapor deposition. The front and back contact electrodes were deposited as AZO transparent conductive films. Then, the mixture containing NaYF_4_:Yb^3+^/Er^3+^ (0.6 wt %) and polymethylmethacrylate (PMMA, 10 wt %) was prepared in trichloroethane. One UC/PMMA layer was spin-coated on the back contact at 900 rpm for 60 s, heated at 120 °C for 1 h and slowly cooled down to room temperature. Finally, white paint was applied as back reflector.

XRD analysis was carried out on a Bruker D8 Advance X-ray diffractometer with Cu Kα radiation, keeping the operating voltage and current at 40 kV and 40 mA, respectively. A 2θ range from 10 to 80° in steps of 6°/min was measured. FE-SEM images and EDX spectroscopy were recorded on Carl Zeiss ULTRA 55 scanning electron microscope at high voltage of 20 kV. Photoluminescence spectra from 500 to 700 nm were recorded with a steady-state & time-resolved fluorescence spectrofluorometer (QM/TM/IM, Photon Technology International) in conjunction with a 980 nm diode laser. The power during measurements was 60 mW. Optical spectra were recorded using a UV–vis–NIR spectrophotometer (Lambda 750S, PerkinElmer) with an integrating sphere (60 mm) attachment. Current–voltage curves were measured by using a solar simulator under standard test conditions (AM1.5, 100 mW/cm^2^ and 25 °C). EQE curves were measured using a QEX10 quantum efficiency measurement system (PV Measurements).
